# Assessing concerns about falling: a comparison of telephone-based and face-to-face FES-I interviews in older adults

**DOI:** 10.1186/s12877-026-08166-3

**Published:** 2026-08-22

**Authors:** Anja Müller, Robert Kob, Ellen Freiberger, Nicolas Rohleder, Sabine Britting

**Affiliations:** 1 Department of Psychology, Chair of Health Psychology, Friedrich- Alexander-Universität Erlangen-Nürnberg, Nägelsbachstrasse 49a, Erlangen, Bavaria 91058 Germany; 2 Department of Internal Medicine-Geriatrics, Institute for Biomedicine of Aging, Friedrich-Alexander-Universität Erlangen-Nürnberg, Kobergerstraße 60, Nürnberg, Bavaria 90408 Germany

**Keywords:** Mode effects, Falls efficacy scale-international, Older adults, Telephone interview, Face-to-face interview, Concerns about falling

## Abstract

**Background:**

Concerns about falling (CaF) are commonly assessed using the Falls Efficacy Scale-International (FES-I), but telephone versus face-to-face interviews may influence responses. The objective of this secondary paired method-comparison analysis was to assess agreement between telephone and face-to-face FES-I total scores in older adults and to explore whether the two administration modes differed at the total-score and item levels.

**Methods:**

Data were drawn from the FEARFALL study, a longitudinal randomized controlled trial on stress and functional health in older adults with CaF. FES-I scores of 123 participants aged 70 years and older were collected via both telephone and face-to-face interviews. Differences in total scores, item-level differences, and regression models were analyzed to identify potential influencing factors.

**Results:**

FES-I scores obtained during the telephone-first assessment were lower than those obtained at the subsequent face-to-face assessment (*p* < .001; *d* = -0.34). Agreement between the two modes was moderate to good (ICC = 0.67). In addition, the two assessment modes showed a high correlation (*r* = .71) with heterogeneity at the item level. Three items showed significant deviations (Item 4: “bathing/showering”, *p*_*adj*_ = 0.007, *d* = 0.36; Item 10: “answer telephone”, *p*_*adj*_ = 0.005, *d* = 0.34; Item 14: “uneven ground” *p*_*adj*_ = 0.015, *d* = 0.32). Age was significantly associated with mode-related score differences (*B* = -0.326, 95% CI [-0.58, -0.07], *p* = .013).

**Conclusion:**

The telephone FES-I may be suitable for group-level comparisons and large-scale screenings. However, the interchangeability of telephone and face-to-face interview for individual clinical use remains uncertain. Since telephone assessment was always performed first and the time interval between assessments varied, the observed differences cannot be attributed to administration mode alone. Further studies specifically designed to compare administration modes are needed.

**Trial registration:**

German Clinical Trials Register (DRKS00029171), registered 22 July 2022.

## Scientific background

Why do some older adults fall a single time, while others fall many times and become more and more immobile, which leads to a poor quality of life [1–3]? This question is difficult to answer since prevalence data on falls vary considerably, mainly due to differences in data collection methods, age thresholds, and gender [4]. However, a systematic review with meta-analysis recently estimates a global fall prevalence of 26.5% among older adults [5]. Falls can not only lead to physical injuries, but may also cause substantial psychological distress [6]. As a consequence, in addition to physical factors, psychological aspects, known as concerns about falling (CaF), increase the risk of further falls [1, 7, 8]. A recent systematic review and meta-analysis revealed that the global prevalence of fear of falling was 49.60% [9]. Xiong et al. (2024) found that prevalence ranged from 6.96 to 90.34%. The subgroup showed that the estimated pooled prevalence of CaF was higher in developing countries (53.40%) than in developed countries (46.7%), and higher in patients (52.20%) than in community residents (48.40%). Overall, a broad range of older adults seem to be affected by CaF.

The terminology used to describe CaF has long been inconsistent. Originally, constructs such as *fear of falling* (FoF) [10], *falls-related self-efficacy* (FSE) [10], *balance confidence* (BC) [11] and *outcome expectancy* (OE) [12] were used. However, the less emotional term *concerns about falling (CaF)* has been established, because it avoids emotional connotations and misunderstandings with anxiety disorders [13]. Therefore, only the term CaF will be used in the following. CaF should be understood multidimensionally and encompasses aspects such as *fear of falling*,* self-efficacy*,* and balance confidenc*e [14]. These factors can manifest as concerns, avoidance of activities, and mobility restrictions. Such restrictions may contribute to a vicious cycle of reduced activity, frailty, further falls, and reduced well-being [1–3, 15].

The World Guidelines for Falls Prevention and Management (WFG) for older adults recommend a multidimensional fall risk assessment that includes balance and gait tests and standardized instruments to assess CaF, such as the Falls Efficacy Scale-International (FES-I) and the Short FES-I [13]. The original Falls Efficacy Scale (FES) [10] was developed to evaluate self-efficacy in everyday activities. However, due to its limitation to simple household activities and its impractical 10-point response format, the European working group ProFaNE (Prevention of Falls network Europe) introduced the FES-I in 2005 as a further development [16]. The revised version includes more complex and social activities, can be used in different cultures, and uses a four-point response format. It has been validated in several European countries including different languages [17, 18]. Studies demonstrated very high internal consistency (Cronbach’s α > 0.90), good test-retest reliability, and convincing construct and criterion validity [17, 19]. In addition, the FES-I has demonstrated its sensitivity to interventions and has now been translated into numerous languages and established internationally [18]. The FES-I is regarded as an essential component of research focusing on CaF and recommended by the WFG [13].

In clinical practice, the FES-I is also recommended as a valuable screening tool for systematically assessing CaF in older adults and identifying those at risk at an early stage [13]. The results obtained with the FES-I support therapy planning, as they provide information on specific needs, such as in the areas of mobility, self-efficacy, or targeted measures for fall prevention [13, 20].

Depending on the setting (e.g. nursing home or community-dwelling) and assessment context, CaF can be measured using self-administered questionnaires, face-to-face interviews or telephone interviews [21–23]. Self-administered questionnaires are a practical, cost-effective, and flexible data collection method. However, they require sufficient understanding of the questions and a certain level of reading ability, which can limit their use, especially among older adults or participants with cognitive impairments [24]. Telephone interviews have been demonstrated to reduce logistical barriers, minimize travel time, and can be implemented cost-effectively in large-scale epidemiological studies [22]. However, they are also vulnerable to distractions in the home environment and may elicit more superficial responses to sensitive questions [22, 25, 26]. Although item wording remains unchanged, differences in administration mode may influence response processes, including interviewer interaction and opportunities for clarification [22, 23]. Conversely, face-to-face interviews permit direct clarification of comprehension problems and closer interaction with older adults, which can yield more precise and nuanced responses [23, 26].

Recent evidence suggests that the mode of data collection can systematically influence responses, depending on question type and individual characteristics, particularly in older adults, for whom memory load and task complexity may play a role [27]. Completing questionnaires at an external location requires more effort, which can be challenging for older adults who are less mobile or anxious [19, 22]. Regardless of the mode of data collection, standardized instructions and trained staff are essential to reduce measurement error and ensure the comparability of results [22].

Several studies have directly compared different modes of data collection. Hauer et al. (2011) found that the interview-administered versions of the FES, FES-I, and Short FES-I were valid and reliable in geriatric populations, including both cognitively intact individuals and those with mild to moderate dementia. Duarte et al. (2023) found an association between telephone interviews and face-to-face interviews in a validation study with older adults, suggesting comparability between both methods. However, systematic reviews indicate that specific effects of mode of data collection may exist at the level of some items, which have not yet been sufficiently investigated [19]. Although the FES-I has been widely studied and applied, the impact of administration mode, particularly telephone versus face-to-face interviews, on response patterns has only been explored to a limited extent. It is conceivable that certain FES-I items are answered consistently across modes, whereas others may be more sensitive to the conditions of data collection. A differentiated analysis at the item level is still pending and represents an unaddressed research gap [22, 23].

The objective of the present study is to examine differences between telephone-based and face-to-face interviews of the FES-I in older adults aged ≥ 70 years. Using data from an existing randomized controlled trial, we explore agreement at the total-score level, examine exploratory item-level differences, and investigate factors associated with mode-related differences.

## Methods

### Study design

This study is based on data from the FEARFALL study, a longitudinal, randomized controlled trial on chronic stress and functional health in older adults with concerns about falling (CaF). The present analyses represent an exploratory secondary analysis of the FEARFALL trial data and were not originally designed to compare FES-I administration modes. The study was pre-registered on the Open Science Framework (OSF) (10.17605/OSF.IO/R4CDX), and the methodological approach, including all questionnaires, is outlined in the study protocol [28]. The Ethics Committee of Friedrich-Alexander University Erlangen-Nuremberg approved the study on August 26, 2020 (protocol 317_20 B), and all participants provided written informed consent. All study procedures adhered to the Declaration of Helsinki. As part of the methodological analysis presented here, we compared two different modes of data collection of the FES-I, face-to-face and telephone interview.

### Participants

The sample included adults living independently, aged 70 years and older. Participants were recruited through a variety of methods, including newspaper advertisements, flyers distributed in public spaces and on public transportation, and direct contact from existing databases. Inclusion criteria were: age ≥ 70 years, a score of ≥ 20 on the 16-item FES-I during telephone screening, independent mobility to the study center, and written consent to participate and be randomized. Exclusion criteria included life expectancy of less than 12 months, previous participation in another intervention study within the past six months, regular use of anti-inflammatory medications (e.g. glucocorticoids or non-steroidal anti-inflammatory drugs [NSAIDs]), and cognitive impairments. Eligible participants were scheduled within eight weeks after screening for a baseline assessment at the study center. Before the baseline assessment, participants received study information and questionnaires to complete at home and bring to the study center. These questionnaires covered demographic, psychological, health-related, and physical activity variables, as described in the study protocol [28]. No study intervention or fall-prevention information was provided during this period, and contact with study personnel was limited to organizational procedures.

### Measures

The measures used in the FEARFALL study are described in detail in the study protocol [28] and preregistration (10.17605/OSF.IO/R4CDX). The following questionnaire is included in the present analysis.

The Falls Efficacy Scale-International (FES-I) assesses CaF and is a standardized tool for evaluating balance and fall-related concerns in clinical and research settings [16]. The 16-item questionnaire was developed specifically for older adults and measures fall-related concerns in various everyday situations. Responses are rated on a four-point scale from 1 (not at all concerned) to 4 (very concerned). The total scores on this scale range from 16 to 64, with higher scores indicating higher CaF. A validated German version has been available since 2006 [29].

The FES-I was administered in two different modes: initially, in a telephone screening to assess eligibility, and subsequently, during the personal baseline examination at the study center, which occurred within eight weeks after screening. Both telephone and face-to-face interviews were conducted by trained members of the FEARFALL study team or trained student assistants using a standardized interview script. All new study personnel received standardized training before conducting interviews. The response options were presented identically in both assessment modes. If clarification was required, interviewers repeated the standardized item wording without providing additional explanations. No visual response aids were used during face-to-face interviews. All trained interviewers could administer either assessment, therefore identity was not systematically recorded. Interviewers conducting the face-to-face assessment had no access to the telephone FES-I score because the data from the two assessments were stored in separate databases and were merged only for the present analysis. The time interval between the telephone-based and face-to-face interview was calculated in days for each participant and summarized as mean (*SD*). This variable “time interval between assessments” was included as a predictor in exploratory regression analyses to account for potential time-related effects.

Other data collected during baseline assessment included the Falls Efficacy Scale-International Avoidance Behavior (FES-IAB) [30] and the Updated Perceived Control of Falling Scale (UP-COF) [31]. Together with the FES-I, these questionnaires were used to obtain a more comprehensive picture of CaF. Furthermore, demographic information (age, sex, body mass index [BMI], residential situation), psychological questionnaires (Perceived Stress Scale (PSS) [32], Geriatric Depression Scale (GDS) [33], Geriatric Anxiety Scale (GAS) [34], Mini Mental State Examination (MMSE) [35]), previous falls and number of falls within the last 12 months, as well as balance and frequency of stumbling, were assessed. The exact dates of reported falls were not recorded. Balance and frequency of stumbling were evaluated subjectively using five- and four-point rating scales, respectively. Balance was rated as *poor*, *fair*, *good*, *very good*, or *excellent*, whereas frequency of stumbling was rated as *never*, *sometimes*, *often*, or *very often*. Physical functionality was measured via the Short Physical Performance Battery (SPPB) [36] and the subjective physical activity level (PA-level).

### Statistics

All analyses were performed in R (Version 2025.09.2 + 418) using the packages psych, irr, dplyr, ggplot2, effsize, broom, and tidyverse. The significance level was set to *α* = 0.05, and all tests were two-tailed. Descriptive statistics, including means, standard deviations, and minimum and maximum values, were calculated for the FES-I scores obtained from the telephone and face-to-face interviews.

Differences between the two modes of data collection at the total score level were examined using paired-samples t-tests, and corresponding effect sizes were calculated. Effect sizes were interpreted according to the conventions proposed by Cohen (2013), where *d* ≈ 0.20 represents a small effect, *d* ≈ 0.50 a medium effect, and *d* ≈ 0.80 a large effect [37]. To assess agreement between the two modes of data collection, Pearson correlations and intraclass correlation coefficients (ICC; two-way mixed model, single measures, absolute agreement) were computed. Following Cicchetti’s (1994) guidelines, ICC values below 0.40 were classified as poor, between 0.40 and 0.59 as moderate, between 0.60 and 0.74 as good, and 0.75 or higher as excellent [38]. Bland-Altman analysis was used to examine systematic differences and limits of agreement. Proportional bias was formally assessed by regressing the paired differences on the paired means.

At the item level, supplementary exploratory analyses included paired-samples t-tests and Cohen’s d, ICC, and Pearson correlations. These analyses were considered hypothesis-generating rather than confirmatory. No additional ordinal or categorical item-level agreement analyses were performed. To control for the increased risk of Type I error due to multiple comparisons, p-values were adjusted using the Bonferroni correction across the 16 items. Bonferroni-adjusted p-values were evaluated at α = 0.05.

Finally, linear regression analyses were conducted to explore factors associated with the difference between telephone and face-to-face total scores. A second exploratory linear regression model examined the association of the telephone FES-I score and the same covariates with the face-to-face FES-I score. Covariates were selected a priori to represent demographic, psychological, fall-related, and functional characteristics available in the FEARFALL dataset that could be associated with FES-I scores or differences between assessment modes. The time interval between assessments was included to examine potential time-related effects. Regression coefficients (*B*), 95% confidence intervals, p-values, R², and adjusted R² were reported. Regression analyses were based on complete-case analysis, no imputation of missing values was performed. Model assumptions were assessed by visual inspection of residual plots, and influential observations were evaluated using Cook’s distance.

Multicollinearity among predictor variables was assessed using variance inflation factors (VIFs). All predictors showed VIF values below 3, indicating no relevant multicollinearity [39].

### AI-assisted copy editing

During manuscript preparation, ChatGPT (GPT-4o; OpenAI, San Francisco, CA, USA) was used to improve readability. The authors reviewed and edited the content as needed and take full responsibility for the final content of the publication.

## Results

The present analyses are based on a sample of 123 participants with complete FES-I data available for both modes of assessment. Of these, 77% were women and 23% men. The average age of the participants was 79.67 years, over half lived alone (58%). Within the last 12 months, 66% reported at least one fall, and 45% of the total sample reported a fall-related injury. Participants had a mean BMI of 26.2 kg/m^2^ and a functional performance (SPPB) averaged 8.8 points. FES-I scores were 28.9 for telephone and 31.0 for face-to-face interviews. The majority of participants rated their balance as fair (59%), and 92% of participants with available data on stumbling (*n* = 86) reported stumbling sometimes or more often (Table 1).

**Table 1:** Description of enrolled older adults

	Descriptive data (*N* = 123)		
	M	SD	range
Age (years)	79.67	5.12	70–91
FES-I (telephone interview)	28.97	6.79	20–46
FES-I (face-to-face interview)	31.02	8.52	20–62
Time interval between assessments (days)	22.38	14.00	1–53
FES-IAB	25.07	7.80	15–56
UP-CoF (*n = 102*)	13.33	4.25	3–20
PSS-10	24.75	7.00	12–43
GAS	14.75	8.25	2–52
MMSE	27.92	1.74	22–30
GDS	2.36	2.29	0–11
BMI	26.23	4.25	19–37
Number of falls (*n = 120*)	1.84	2.99	0–25
SPPB (*n = 122*)	8.79	2.35	2–12
PA-level	1.92	0.70	1–3

		**Frequency**	
		(*N* = 123)	
		absolute	percent
Sex	Female	95	77
	Male	28	23
Residential situation	Living alone	71	58
	Not living alone	52	42
Previous falls (yes/no)	≥ 1 within the last 12 months	81	66
	Injuries	55	45
Balance	Poor	38	31
	Fair	73	59
	Good	11	9
	Very good	1	1
	Excellent	0	0
Frequency of stumbling (*n = 86*)	Never	7	8
	Sometimes	65	76
	Often	10	12
	Very often	4	5

*M *mean; *SD S*tandard deviation; *FES-I* Falls efficacy scale-international; *FES-IAB* Falls efficacy scale-international avoidance behavior; *UP-CoF *Updated perceived control over falling scale; *PSS-10 *Perceived stress scale 10; *GAS *Geriatric anxiety scale; *MMSE *Mini-mental state examination; *GDS *Geriatric depression scale; *BMI *Body mass index; *SPPB* Short physical performance battery; Sex was coded as 1 = female and 2 = male, Previous falls were coded as 1 = yes and 2 = no; Balance was coded from 1 = poor to 5 = excellent; Frequency of stumbling was coded from 1 = very often to 4 = never

**Fig. 1:**
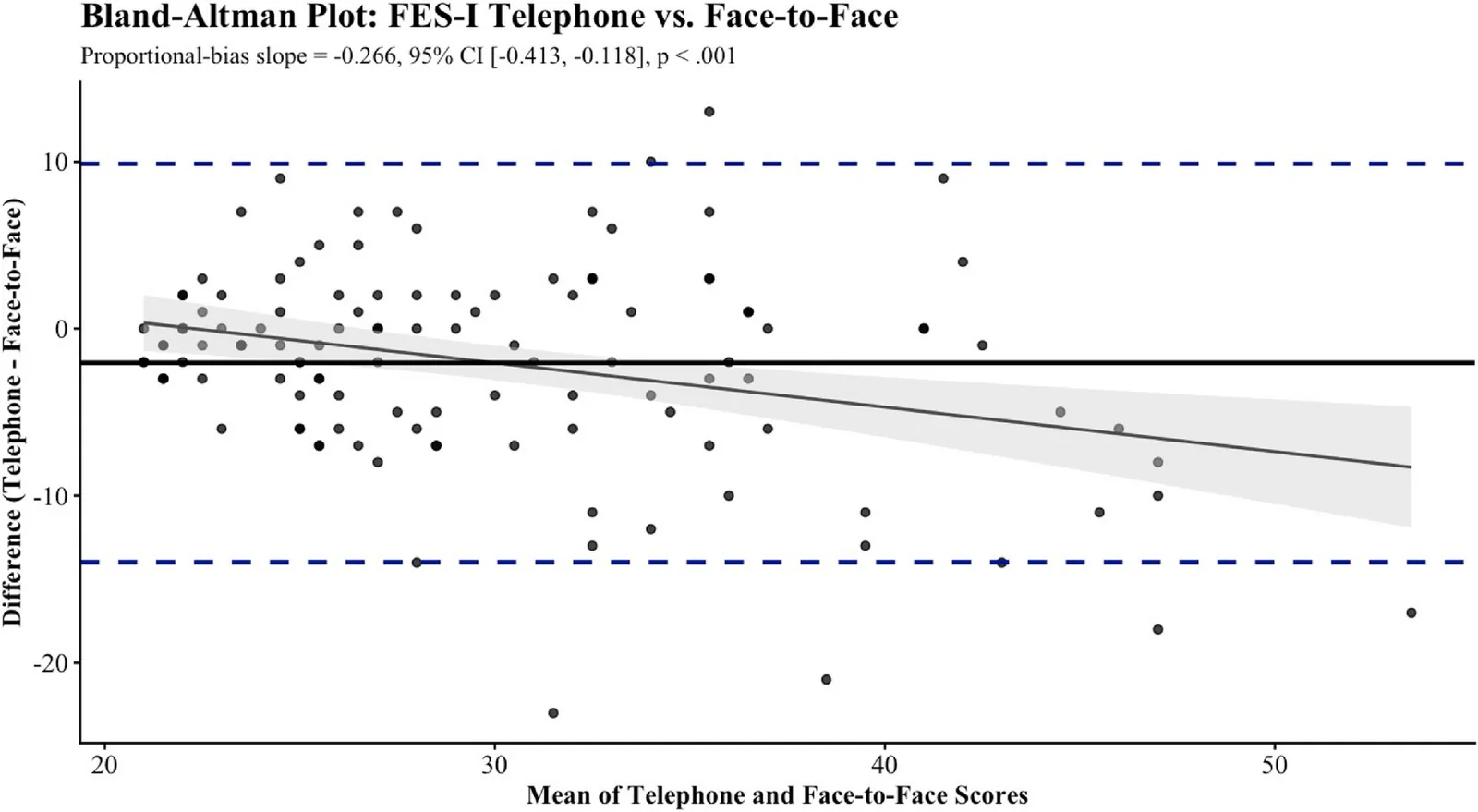
Bland-Altman plot comparing FES-I scores obtained via telephone and face-to-face interviews

Descriptive analyses showed that FES-I scores were higher in the face-to-face interview (*M* = 31.02, *SD* = 8.52) than in the telephone interview (*M* = 28.97, *SD* = 6.79). A Bland-Altman analysis revealed a mean bias of -2.05 (95% CI [-3.14, -0.96]), indicating that telephone interviews tended to yield slightly lower scores than face-to-face interviews. The limits of agreement (LoA) ranged from − 13.98 (95% CI [-15.84, -12.12]) to 9.88 (95% CI [8.02, 11.74]). Regression of the paired differences on the paired means indicated evidence of proportional bias (slope = -0.266, 95% CI [-0.413, -0.118], *p* < .001), suggesting that differences between administration modes became larger with increasing FES-I scores (Fig. 1).

A t-test for paired samples showed that this difference was statistically significant *t*(122) = -3.73, *p* < .001, with a small effect size of *d* = -0.34. A sensitivity analysis using the Wilcoxon signed-rank test yielded comparable results (*p* < .001). The intraclass correlation (ICC) was 0.67, 95% CI [0.53, 0.76], corresponding to moderate to good agreement according to the prespecified Cicchetti (1994) classification. Pearson’s correlation coefficient was 0.71, indicating a strong positive association between telephone and face-to-face FES-I scores.

**Fig. 2:**
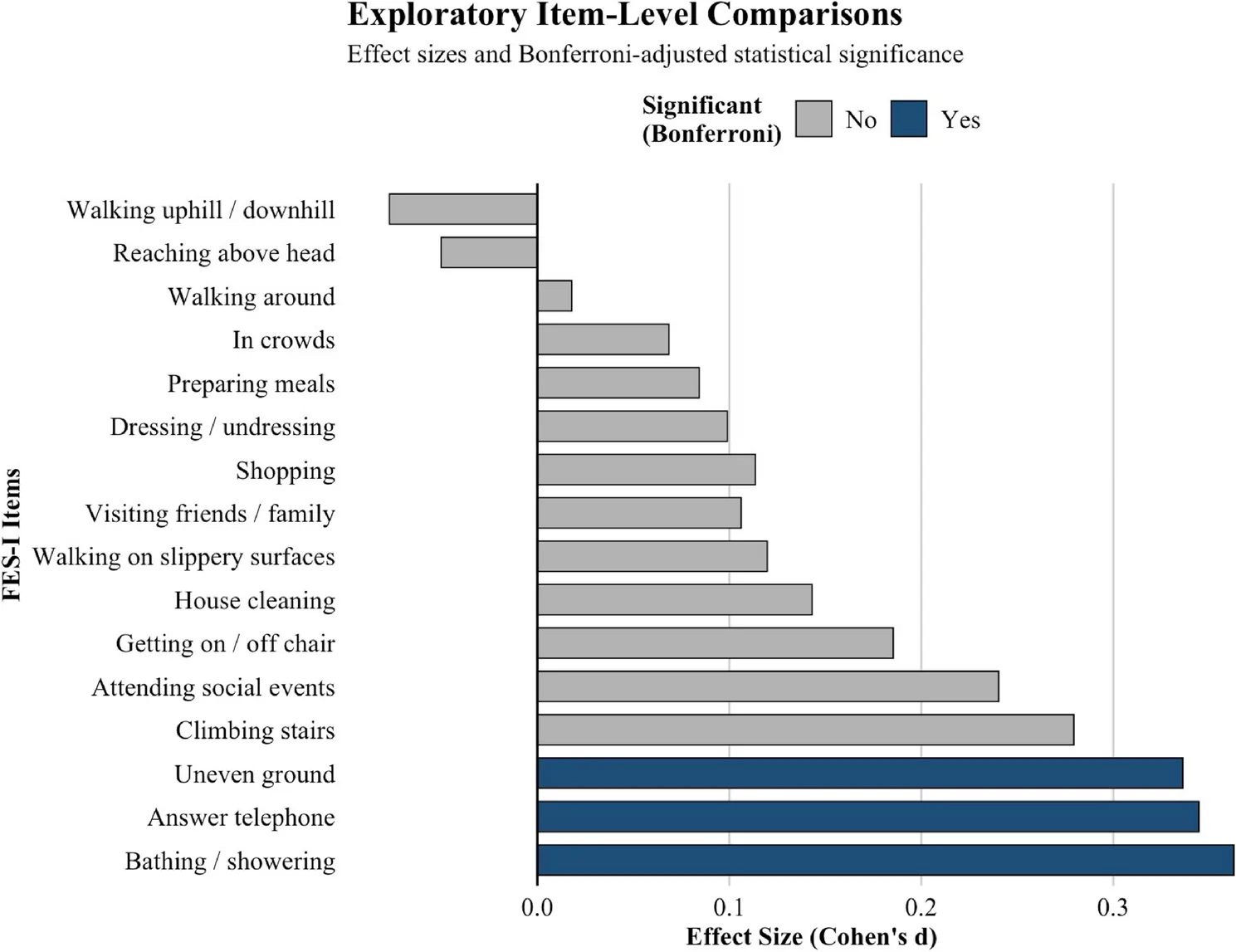
Item-wise comparison of the FES-I between telephone- and face-to-face interviews. Effect sizes (Cohen’s d) are shown; significant differences after Bonferroni correction are marked in dark blue; positive d-values indicate higher scores in the face-to-face interview compared to the telephone interview; negative d-values indicate higher scores in the telephone interview

In order to examine the differences between the telephone and face-to-face interviews in more detail, an exploratory item analysis of the FES-I was conducted. Paired t-tests were performed for each item, using the Bonferroni correction to control for Type I error in multiple comparisons (Table 2). The majority of items exhibited no significant deviations between telephone and face-to-face interviews. After Bonferroni correction, three items differed significantly: Item 4 *Bathing / showering* (*t*(122) = -3.60, *p*_*adj*_ = 0.007, *d* = 0.36), Item 10 *Answer telephone* (*t*(122) = -3.72, *p*_*adj*_ = 0.005, *d* = 0.34), and Item 14 *uneven ground (t*(122) = -3.40, *p*_*adj*_ = 0.015, *d* = 0.32). Other items showed only marginal deviations (*d* ≈ 0.20 and smaller), indicating small effects [37] and were considered non-significant following correction (Fig. 2).

**Table 2:** Exploratory item-level comparisons of telephone- and face-to-face interview

Item Label	*M*_*Tel*_*(SD*)	M_F2F_ (*SD*)	Δ *M*	ICC [95% CI]	*r*	*p*	*p*_adj_
Total Score	28.97 (6.79)	31.02 (8.52)	-2.05	0.67 [0.53, 0.76]	0.71	**< ** **0.001**	
1. House cleaning	1.50 (0.87)	1.63 (0.94)	-0.13	0.46 [0.31, 0.59]	0.47	0.128	1.000
2. Dressing / undressing	1.91 (1.05)	2.02 (1.09)	-0.11	0.46 [0.31, 0.59]	0.46	0.292	1.000
3. Preparing meals	1.07 (0.32)	1.11 (0.44)	-0.03	0.17 [-0.01, 0.34]	0.18	0.467	1.000
4. Bathing / showering	2.13 (1.05)	2.51 (1.06)	-0.38	0.35 [0.19, 0.50]	0.38	< 0.001	**0.007**
5. Shopping	1.29 (0.65)	1.37 (0.77)	-0.08	0.27 [0.09, 0.42]	0.27	0.299	1.000
6. Getting on / off chair	1.30 (0.60)	1.43 (0.78)	-0.13	0.34 [0.17, 0.48]	0.35	0.074	1.000
7. Climbing stairs	2.67 (0.97)	2.93 (0.95)	-0.27	0.39 [0.23, 0.53]	0.40	0.005	0.085
8. Walking around	1.64 (0.86)	1.66 (0.95)	-0.02	0.35 [0.19, 0.50]	0.35	0.862	1.000
9. Reaching above head	1.94 (0.89)	1.89 (1.04)	0.05	0.46 [0.31, 0.59]	0.46	0.592	1.000
10. Answer telephone	1.38 (0.73)	1.68 (0.97)	-0.30	0.43 [0.27, 0.57]	0.47	< 0.001	**0.005**
11. Walking on slippery surfaces	3.20 (0.84)	3.30 (0.79)	-0.10	0.39 [0.23, 0.53]	0.39	0.232	1.000
12. Visiting friends / family	1.33 (0.67)	1.41 (0.84)	-0.08	0.52 [0.38, 0.64]	0.53	0.227	1.000
13. In crowds	1.76 (1.05)	1.83 (1.08)	-0.07	0.43 [0.28, 0.57]	0.43	0.478	1.000
14. Uneven ground	2.37 (0.94)	2.69 (0.95)	-0.32	0.38 [0.21, 0.52]	0.40	0.001	**0.015**
15. Walking uphill / downhill	2.15 (1.00)	2.07 (1.10)	0.08	0.37 [0.20, 0.51]	0.37	0.448	1.000
16. Attending social events	1.28 (0.65)	1.47 (0.87)	-0.19	0.37 [0.21, 0.51]	0.40	0.016	0.263

*FES-I *Falls efficacy scale-international; *M*_*Tel*_ = mean telephone interview; *M*_*F2F*_ = mean face-to-face interview; *Δ M* = paired mean difference (telephone − face-to-face); *ICC* Intraclass correlation coefficient; *CI* Confidence interval; *r* Pearson correlation; *p* p-value; p-values are based on paired t-tests (α = 0.05); *p*_adj_= p-values are based on paired t-tests and were adjusted using Bonferroni correction for multiple comparisons; Item-level analyses were exploratory and hypothesis-generating; No ordinal or categorical item-level agreement statistics were performed

Bold values indicate significance after Bonferroni correction at the 5% level

To investigate potential factors influencing the difference between the FES-I total scores obtained by telephone- and face-to-face interview, a linear regression analysis was performed. The regression analyses were based on 79 complete cases. The dependent variable was defined as the difference between the FES-I scores (telephone – face-to-face). Age, sex, BMI, MMSE, GDS, PSS, GAS, previous falls, number of falls, balance, frequency of stumbling, time interval between assessments and SPPB were included as predictors. All predictors showed VIF values below 3, indicating no relevant multicollinearity. Visual inspection of the residual plots did not indicate substantial violations of the regression assumptions, and no influential observations were identified based on Cook’s distance. The analysis showed that only age was a significant predictor of differences between the modes of data collection (*B* = -0.326, 95% CI [-0.58, -0.07], *p* = .013). With increasing age, the FES-I score obtained by telephone tended to be lower than the face-to-face score. No other predictors were significant. The model had an *R²* of 0.275 and an *adjusted R²* of 0.130 (Table 3).

**Table 3:** Influence of covariates on FES-I score difference

Characteristic	B	95% CI	*p*
Age	-0.326	[-0.58; -0.07]	**0.013**
Sex	1.941	[-1.40; 5.28]	0.250
BMI	0.087	[-0.25; 0.42]	0.603
MMSE	0.094	[-0.64; 0.83]	0.798
GDS	-0.351	[-1.00; 0.30]	0.285
PSS-10	0.167	[-0.10; 0.44]	0.222
GAS	-0.165	[-0.39; 0.07]	0.157
Previous falls	-0.608	[-4.07; 2.85]	0.727
Number of Falls	-0.248	[-1.08; 0.59]	0.556
Balance	0.987	[-1.14; 3.11]	0.357
Frequency of stumbling	0.779	[-1.40; 2.95]	0.477
SPPB	0.279	[-0.34; 0.90]	0.372
Time interval between assessments	0.043	[-0.04; 0.12]	0.303
*R²*	0.275		
*adjusted R²*	0.130		

*B *Unstandardized regression coefficient; *CI *Confidence interval; *BMI *Body mass index; *MMSE *Mini-mental state examination; *GDS *Geriatric depression scale; *PSS-10 *Perceived stress scale 10; *GAS *Geriatric anxiety scale; *SPPB *Short physical performance battery; Dependent variable: difference between telephone and face-to-face FES-I scores (telephone – face-to-face)

Bold values indicate significance at the 5%level

Furthermore, a regression analysis was used to investigate predictors of the results of the face-to-face interview (Table 4). Telephone interview proved to be a significant predictor of the total face-to-face score (*B* = 0.805, 95% CI [0.61, 1.00], *p* < .001). Another significant predictor was age (*B* = 0.349, 95% CI [0.10, 0.6], *p* = .007). The results showed that there were no significant effects for sex, BMI, MMSE, GDS, PSS, GAS, previous falls, number of falls, balance, frequency of stumbling, time interval between assessments and SPPB (*p* > .05). All predictors showed VIF values below 3, indicating no relevant multicollinearity. The model had an *R²* of 0.709 and an *adjusted R²* of 0.646.

**Table 4:** Exploratory linear regression of face-to-face FES-I score on telephone FES-I score and covariates

Predictor	B	95% CI	*p*
FES-I telephone	0.805	[0.61; 1.00]	**< ** **0.001**
Age	0.349	[0.10; 0.60]	**0.007**
Sex	-1.704	[-4.98; 1.57]	0.302
BMI	-0.080	[-0.41; 0.25]	0.626
MMSE	0.049	[-0.68; 0.78]	0.894
GDS	0.371	[-0.26; 1.01]	0.247
PSS-10	-0.123	[-0.39; 0.15]	0.363
GAS	0.167	[-0.06; 0.39]	0.142
Previous falls	0.679	[-2.70; 4.06]	0.690
Number of Falls	0.218	[-0.60; 1.03]	0.595
Balance	-0.972	[-3.05; 1.11]	0.354
Frequency of stumbling	-1.367	[-3.57; 0.83]	0.219
SPPB	-0.368	[-0.98; 0.24]	0.234
Time interval between assessments	-0.034	[-0.11; 0.05]	0.410
*R²*	0.709		
*adjusted R²*	0.646		

*B *Unstandardized regression coefficient; *CI *Confidence interval; *BMI *Body mass index; *MMSE *Mini mental state examination; *GDS *Geriatric depression scale; *PSS-10 *Perceived stress scale 10; *GAS *Geriatric anxiety scale; *SPPB *Short physical performance battery

Bold levels indicate significance at the 5% level

## Discussion

The aim of this analysis was to examine different modes of administering the Falls Efficacy Scale-International (FES-I) for assessing concerns about falling (CaF) in older adults. For this purpose, the total scores of the telephone- and the face-to-face interviews were compared. In both clinical practice and research, remote and telephone-based assessments have become increasingly common. These assessment modes offer several advantages, including flexibility and cost-efficiency, as they can be conducted independently of time and location. These factors also gained particular relevance during the COVID-19 pandemic [40, 41]. Current research provides only limited evidence for the validity of telephone interviews as an instrument for adequately assessing CaF in people over 70 years [22]. According to the existing literature, it was hypothesized that there would be no significant differences between the two modes of data collection and that their results would show a good to excellent level of agreement [22]. This hypothesis was not supported by our findings. A statistically significant discrepancy was identified in the total scores (telephone interview vs. face-to-face interview). Furthermore, three items differed significantly between administration modes (Items 4, 10, and 14). At the individual level, there is poor to moderate agreement across items. Exploratory analyses indicated that age was significantly associated with the difference between telephone and face-to-face total scores and with the face-to-face score.

### Large-scale screening versus individual diagnostics

The analysis showed that the mean values of the telephone interview were significantly lower than those of the face-to-face interview, although the effect size was small. The results revealed a strong linear association between the two assessment modes and a moderate to good level of agreement at the group level according to the ICC. However, the Bland-Altman analysis indicated proportional bias, suggesting that differences between administration modes became more pronounced at higher average FES-I scores. These findings indicate limited comparability between the modes. Our findings differ from the analyses of Duarte et al. (2023), who identified a high degree of consistency between telephone and face-to-face interviews in a Brazilian sample. Based on their results, Duarte et al. (2023) concluded that the telephone interview is an equivalent alternative to face-to-face interviews. In contrast, the present analysis indicates only partial agreement, suggesting limited transferability to vulnerable populations and people over 70 years.

The divergent results can be attributed to differences in study conditions. The sample in the present study was older on average and consisted solely of participants with existing CaF (FES-I ≥ 20), whereas Duarte et al. (2023) also included participants without CaF. Age-related limitations were likely more prevalent in the current sample. Furthermore, the study by Duarte et al. (2023) was conducted in a controlled research setting, with a maximum interval of one week between data collection. The present study had a time gap of 1 to 53 days between assessments, although variability in the time interval was explicitly accounted for in the analyses, which only partially addresses potential time-related changes. True changes in CaF between assessments cannot be fully excluded. Therefore, the observed differences cannot be attributed solely to the mode of administration.

In addition to the different time and methodological aspects as shown in the study by Duarte et al. (2023), other factors may also play a role. One possible explanation is that age-related changes in functioning also contributed to the observed FES-I score differences. However, sensory impairments were not systematically assessed in the present study and therefore cannot be directly examined. Age-related hearing impairment and reduced efficiency in processing information without visual cues may make it more difficult for older adults to follow telephone-administered instructions. Another relevant factor is the lack of nonverbal communication [42]. Nonverbal signals, empathic resonance, and situational cues are more present in face-to-face interactions and can intensify emotional responses to questions [26, 42]. It is possible that the anonymity of telephone interviews encourages participants to disclose their concerns or fears more openly, while the emotional distance reduces affective involvement. The absence of nonverbal communication may thus partly explain the lower scores observed in telephone interviews.

### Age-related differences

The present analyses also indicated an exploratory association between age and the difference between telephone interviews and face-to-face interviews. With increasing age, the difference between telephone and face-to-face FES-I scores became more negative. Older age was associated with larger differences between the two assessments. It extends the observations of Duarte et al. (2023), who did not find any age-dependent mode effects in a sample that was approximately 10 years younger. The results suggest that the comparability of telephone and face-to-face interviews may be limited at the individual level among older adults. Although Duarte et al. (2023) indicate that the FES-I telephone interview is a reliable method for assessing CaF, the observed mode-related differences suggest that classification around the FES-I cut-off value used for eligibility may differ depending on the administration mode. However, because all participants were selected based on a telephone FES-I score of ≥ 20, possible differences in classification could not be directly evaluated. In summary, the observed level of agreement between the two modes of data collection supports their comparability at the group level, whereas their interchangeability for individual assessments remains uncertain.

### Item-specific differences

While Duarte et al. (2023) reported that differences between modes at the item level were minor, the present analysis suggests that certain items are more context-sensitive in populations of older adults. Significant differences were found for three items: “bathing / showering”, “answer telephone”, and “uneven ground”. Activities such as bathing or showering and walking on uneven ground require awareness of physically demanding or potentially risky situations. Given age-related changes in cognitive processes [43], such as executive functions, these activities may be appraised differently across administration modes. In face-to-face interviews, these situations may be perceived as more concrete and physically challenging, whereas telephone assessments may encourage a more abstract evaluation. For activities related to personal hygiene, feelings of shame or intimacy may additionally play a role, as such concerns may be easier to communicate in direct and personal contact where trust and empathy can develop [26, 42].

The item “answer telephone” represents a situation that is characterized by time pressure and the need for rapid movements in response to an external stimulus. In face-to-face interviews, this scenario may be perceived as more concrete and physically demanding, as the situational context and physical requirements are more prominent. In contrast, telephone assessments may encourage a more abstract assessment of the situation, which could potentially lead to an underestimation of the perceived fall risk. This difference in situational vividness may partly explain the lower scores observed for this item in telephone interviews. When imagining various activities, cognitive abilities such as planning, problem solving, and visualization appear to be more important. On the telephone, item-specific visual or nonverbal cues are unavailable, which may lead to an underestimation of the potential challenge.

These item-level findings may reflect two possible mechanisms for mode effects: (1) social presence, which can enhance openness and emotional involvement through empathetic interaction and a protected environment, and (2) cognitive demands in an auditory-only setting, which may make it more difficult to imagine specific situations. Existing literature on older adults indicates mode effects, but it focuses more on differences in task execution than on their underlying causes [44].

### Clinical and methodological implications

From a clinical perspective, the present findings are of practical relevance. Guidelines confirm the suitability of the FES-I as an instrument for assessing CaF [13]. The observed differences suggest that further research is needed to examine whether assessment sequence or administration mode may affect the identification of vulnerable individuals or subsequent clinical decisions. In contrast to the recommendations of Duarte et al. (2023), the interchangeability of telephone and face-to-face interview at the individual level remains uncertain, particularly in older and vulnerable populations. Although telephone interviews may provide sufficiently reliable results for group comparisons, screenings, and epidemiological analyses, they should be interpreted with caution when used as a sole basis for clinical decisions in individual cases. Additionally, age was associated with larger mode-related score differences. Future studies should examine whether supplementary procedures, such as combined screening approaches or standardized adaptations of interview procedures, may improve comparability between assessment formats in older adults. The present analysis underscores the importance of carefully considering the mode of data collection in validation studies, particularly for questionnaires addressing emotional or context-dependent topics. Findings from younger populations, such as those by Duarte et al. (2023) should therefore be generalized to older adults or vulnerable individuals only with caution.

### Strengths and limitations

The strengths of this analysis include the within-subject design, allowing direct comparison of modes of data collection (telephone vs. face-to-face) and eliminating interindividual variability. Moreover, the analyses controlled for a broad range of demographic, psychological, and functional covariates. This allowed for a more detailed examination of potential factors associated with mode-related differences.

Additionally, the study focused on a clinically relevant target group that is important for fall prevention and intervention. Compared to previous studies, such as Duarte et al. (2023), the higher mean age of the present sample can be considered a strength, as it reflects a population in which CaF assessments are particularly relevant.

An important limitation of the present analysis is the non-standardized time interval between the two assessments. Although the assessments were not conducted within a standardized short test–retest interval, variability in the time interval between assessments was explicitly quantified and considered in exploratory analyses to partially account for potential time-related changes. Nevertheless, changes in CaF between assessments cannot be fully ruled out. Given the time interval and the fixed order of assessments, the present secondary analysis cannot distinguish administration-mode effects from changes over time, order effects, or effects of repeated assessment.

Furthermore, the present analysis represents an exploratory secondary analysis of data from a randomized controlled trial that was not primarily designed to compare modes of FES-I administration. Because eligibility was based on the telephone FES-I score (≥ 20), potential selection bias, range restriction, and regression-to-the-mean effects cannot be excluded and should be considered when interpreting the findings. The regression analyses were additionally based on 79 complete cases, excluding 44 participants with missing covariate data. This complete-case selection may have introduced further bias. Some variables that may be relevant for interpreting mode effects were not systematically collected. In particular, hearing and other sensory impairments were not systematically assessed in the present study. Given that some FES-I items involve time pressure or rely on auditory cues, such impairments could potentially influence responses in telephone-based assessments. However, their impact cannot be directly evaluated within the current dataset. Interviewer identity was not systematically recorded, so potential interviewer effects and whether the same interviewer conducted both assessments could not be examined.

Finally, the analyzed participants are not representative of the general older population. Only participants with at least moderate CaF (FES-I ≥ 20) were included, participants without concerns were not represented. Particularly, persons were recruited that either had experiences in clinical studies or who independently contacted our study team after reading the advertisement searching for persons with CaF. Individuals who volunteered or responded to study advertisements may be more willing to communicate concerns or fears than others, which may limit generalizability. Furthermore, the findings may not generalize to institutionalized older adults or to individuals with severe frailty, cognitive impairment, or significant hearing impairment.

## Conclusion

The present findings suggest that telephone-based interviews of the FES-I yield systematically lower scores than face-to-face interviews in older adults. While telephone interviews appear suitable for group-level research and large-scale screening, the observed discrepancies, particularly among adults aged 70 years and older, suggest caution when interpreting individual scores, especially around established FES-I cut-off values. Therefore, the interchangeability of the two administration modes for individual clinical use remains uncertain. Because administration mode and time were not separable in the present design, these findings require confirmation in studies specifically designed to compare administration modes.

Future studies should further investigate the mechanisms underlying mode effects, such as cognitive load and social presence, sensory impairment (e.g. hearing) and evaluate adaptive strategies to improve comparability between screening formats. Combining modes or adapting communication to age-related needs may help enhance accuracy and inclusivity in the assessment of CaF among older adults.

## Data Availability

Due to privacy restrictions, de-identified participant data are not publicly available. Data may be made available from the corresponding author upon reasonable request and subject to approval by the ethics committee and a data use agreement. Analysis code, statistical syntax, and a data dictionary may also be provided upon reasonable request. Upon completion of the study and finalization of the primary analyses, de-identified data will be made available through a public repository.

## Abbreviations

BMI: Body mass index.
BC: Balance confidence.
CaF: Concerns about falling.
CI: Confidence interval.
FES-I: Falls efficacy scale-international.
FES-IAB: Falls efficacy scale-international avoidance behavior.
FoF: Fear of falling.
FSE: Falls-related self-efficacy.
GAS: Geriatric anxiety scale.
GDS: Geriatric depression scale.
ICC: Intraclass correlation coefficient.
LoA: Limits of agreement.
MMSE: Mini mental state examination.
OE: Outcome expectancy.
OSF: Open science framework.
PA-level: Physical activity level.
PSS: Perceived stress scale.
ProFaNE: Prevention of falls network europe.
SPPB: Short physical performance battery.
UP-COF: Updated perceived control of falling scale.
VIF: Variance inflation factors.
WFG: World falls guidelines.
